# Psychometric Characteristics of the Persian (Farsi) Version of Attachment Style Questionnaire

**Published:** 2014-11

**Authors:** Ali Firoozabadi, Zabihollah Abedi, Roqayeh Aliyari, Behzad Zolfaghari, Ahmad Ghanizadeh

**Affiliations:** 1 Research Center for Psychiatry and Behavioral Sciences, Department of Psychiatry, School of Medicine, Hafez Hospital, Shiraz University of Medical Sciences, Shiraz, Iran;; 2 School of Public Health, Shahroud University of Medical Sciences, Shahroud, Iran;; 3 Department of Pharmacognosy, School of Pharmacy and Pharmaceutical Sciences, Isfahan University of Medical Sciences, Isfahan, Iran

**Keywords:** Iran, Questionnaire, Culture

## Abstract

**Background: **Attachment relationship provides a secure base for the infants from which to explore the environment and a safe haven to return to in times of danger. Attachment style shapes the behavior of individuals in adulthood. There are many different measures of attachment and a lot of controversy about what they measure and how they relate to each other. Hence, we tried to evaluate the psychometric properties of one of such questionnaires on a sample of the Iranian population.

**Methods: **“Attachment style questionnaire” designed by Van Oudenhoven measures four dimensions: secure, preoccupied, fearful and dismissing. Psychometric properties of the questionnaire were evaluated in a cross sectional study on 730 adults in Isfahan, Iran. Statistical analysis of data was performed by the explanatory factor analysis with the principal component method, Cronbach’s alpha, Pearson correlation coefficients, and the multiple analysis of variance (MANCOVA).

**Results:** The Cronbach’s alpha for all items was 0.704. As a whole, the internal consistency was good. There was a high inter-scale correlation between preoccupied and fearful, also the secure style correlated negatively with fearful and preoccupied. The stability coefficient of the attachment scales were 0.625, 0.685, 0.777 and 0.605 for secure, fearful, preoccupied and dismissing styles respectively (P<0.001). Regarding construct validity, factor analysis showed that some items require iterations to fit the Iranian population.

**Conclusion:** This study showed that the Persian version of ASQ has a reasonable reliability and validity in general and the questionnaire is appropriate for use among the Iranian population in future studies.

## Introduction

Attachment can be defined as “a continuous tie to a specific person that a child turns to when feeling vulnerable and in need of protection”.^1^

According to the attachment theory, experiences resulted from previous attachments to significant others are internalized to form cognitive structures, or working models, that shape individuals’ expectations of and beliefs about the past, present, and future social interactions. Defining individual differences in attachment in terms of the intersection of two dimensions, namely a model of self and others, Bowlby systematized his conception of the internal working models.^2^^-^^4^ Ainsworth in her work on “strange situation” showed that when the caregiver consistently responds to child’s need, a fundamental trust and a secure attachment would be developed between a child and his/her caregiver. In contrast, in the absence of responsiveness and availability of caregiver, two insecure attachment patterns would be formed, including an anxious-ambivalent or avoidant attachment pattern.^5^

In the 1980s, George, Kaplan, and Main developed the Adult Attachment Interview (AAI) to assess attachment in adults.^6^ Hazan and Shaver evaluated the three attachment patterns in adults by asking individuals to classify themselves based on the description presented in three separate statements.^7^^,^^8^ Bartholomew & Horowitz constructed a four-category model of adult attachment.^9^ In this model, they separated the avoidant attachment pattern into two categories.

Today, as Crowell et al. noted, there are many different measures of attachment and also a great deal of confusion about what they measure, what they are supposed to measure, and how they are related to each other.^10^ Some researchers are in favor of the universality of attachment theory.^11^^-^^13^ However, few studies point out the role of cultural difference in attachment styles.^14^^-^^17^ In western culture secure attachment is expressed by individuality, self-confidence and independence. However, qualities such as interdependency, relationship with family members and self-denial are important issues in eastern cultures. Several tools, mostly in the form of questionnaires, have been designed to measure and evaluate people’s attachment styles. Hazan and Shaver’s^7^ self-report measure was among the first. Based on this measure the Relationship Questionnaire^9^^,^^18^ was developed. These measures used just one item and determination of their internal reliability was impossible. Different researchers have divided the items into several phrases so that they could be scored as items on a Likert scale. Similar to Hazan and Shaver’s vignettes, Simpson,^19^ Collins and Read,^20^ designed multiple-item questionnaires. Griffin and Bartholomew^21^ developed the Relationship Scales Questionnaire (RSQ) based on the relationship questionnaire of Bartholomew and Horowitz.^9^ Feeney et al. developed a measure based on Bartholomew’s four-type attachment styles for use with adolescents and others who have had little experience with an intimate relationship outside the family.^22^^,^^23^ A rather recent measure is the one designed by Van Oudenhoven et al. which we used in this study.^24^ This instrument (ASQ) measures four adult attachment styles, based on the theoretical model of Bartholomew and the RSQ of Griffin and Bartholomew. The current study assesses the psychometric properties of this questionnaire on a sample of the Iranian population. In addition, we compared attachment styles according to variables such as sex, level of education, level of parent’s education, birthplace, and marital status.

## Materials and Methods

In this study, we recruited adult population aged 18 to 65 with at least an 8^th^ grade education. They were selected from Isfahan by first order clustering. In each cluster, individuals were chosen randomly based on geographical region as assigned by the governmental health organization. Seven centers were randomly selected and 800 questionnaires were distributed from which 750 questionnaires were completed. Ten questionnaires were excluded due to multiple missing values. Moreover, Mahalanobis distance was used to identify unsuitable questionnaires and consequently ten other questionnaires with the highest Mahalanobis distances were omitted. Eventually, 730 questionnaires were selected for the inclusion in the study.

Linguistic validation: based on the standard forward-backward methodology, the questionnaire was initially evaluated and translated into Persian. After editing this initial translation, it was back translated to English, and the final questionnaire was developed by comparing the two English versions of the questionnaires. The draft questionnaire was examined in a pilot study using 70 individuals. The final version of the questionnaire was then prepared according to the outcome of this pilot study. 

*Instruments*

ASQ: Attachment Style Questionnaire (ASQ)^24^ includes 24 items that generally deal with attachment to others. Items were constructed based on the four vignettes established by Bartholomew and Horowitz^2^ and the Relationship Scales Questionnaire designed by Griffin and Bartholomew.^21^ Among all questions, seven items measured the secure style, five items dealt with the scale for fearful attachment, seven items indicated preoccupied style and finally, dismissing scale involved five items. All attachment items were measured according to a 5-point Likert scale, from 1 (strongly disagree) to 5 (strongly agree). Two items were mirrored. The Cronbach’s alpha coefficients of the subscales were 0.73, 0.75, 0.80, and 0.61 for secure, fearful, preoccupied, and dismissing styles respectively.^24^

RQ (Relationship Questionnaire): the reliability and validity of the Relationship Questionnaire (RQ) of Hazan and Shaver^7^ among the Iranian population were assessed by Pakdaman et al.^25^ The total Cronbach’s alpha was 0.789. RQ consists of three short paragraphs, which are measured in two parts; in the first part, each sentence was rated in the 7-point Likert-type and in the second part, each individual chose just one paragraph that was closer to his/her attachment style. We used RQ to assess convergent validity.

Feeney’s Attachment Style Questionnaire: this questionnaire includes 40 items, which are constructed to measure five factors (confidence in self and others, discomfort with closeness, relationships as secondary, preoccupation with relationships and the need for approval scales). Each item is rated on a 6-point Likert response ranging from 1 (completely disagree) to 6 (completely agree). The reliability and validity of the Persian version of the questionnaire have been assessed previously. Cronbach’s alpha for the Confidence, Need for approval, Preoccupation, Relationship as secondary and Discomfort styles were calculated to be 0.515, 0.588, 0.752, 0.532 and 0.639 respectively.^22^^,^^23^ This questionnaire is also used to assess convergent validity.

Reliability: to determine the reliability by test-retest method, we redistributed the questionnaire amongst 98 individuals of the initial samples, three months after the first sampling. In addition to Cronbach’s alpha, we used Cicchetti’s guideline^26^ to interpret the internal consistency coefficients (>0.4 poor, 0.41-0.59 fair, 0.6-0.74 good, >0.75 excellent)

To assess construct validity, the explanatory factor analysis with the principal component method and quartimax rotation (EFA) was performed. Our Statistical criteria were; the scree plot, eigenvalues greater than 1.2, percentage of explained variance and component loadings greater than 0.3.^27^

The Pearson correlation coefficient was used on both Feeney’s questionnaire and RQ to assess the convergence validity. By the multiple analysis of covariance (MANCOVA), we evaluated the relationship between the ASQ domains (secure, fearful, preoccupied, and dismissing) and variables such as sex, birthplace, education levels, parents’ education levels, and marital status. 

*Statistical Analysis*

All data were analyzed by the statistical package for social sciences (SPSS V.19) software. Statistical analysis of data was performed by the explanatory factor analysis with the principal component method and quartimax rotation, Cronbach’s alpha and Pearson correlation coefficients and the multiple analysis of variance (MANCOVA)*.*

## Results

730 subjects (475 women and 255 men) participated in the study, the mean age of participants was 29.5 years (SD=9.74); 132 (18.1%) participants had less than twelve years of education and 349 (47.8%) had graduated from high school. The others had higher education. 654 (89.5%) were born in urban areas and 368 (50.4%) were married. 

*Reliability*

Internal consistency: the Cronbach’s alpha coefficients for the four scales are presented in table 1. Item 7 in secure subscale and item 15 in preoccupied subscale were recorded by inverse score. The Cronbach’s alpha for all items was 0.704.

**Table 1:** Cronbach’s alpha coefficient for the four scales of ASQ-24

**Scales**	**Secure**	**Fearful**	**Preoccupied**	**Dismissing**
Cronbach’s alpha	0.630	0.766	0.723	0.588

The inter-scale correlation is represented in table 2. There is a high correlation between preoccupied and fearful styles, also the secure style correlated negatively with fearful and preoccupied styles.

**Table2:** Interscale correlation coefficients

	**Fearful **	**Preoccupied**	**Dismissing**
Secure	-0.192^*^	-0.036	0.198^*^
Fearful	—	0.349^*^	0.288^*^
Preoccupied	—	—	-0.070

Stability: a group of participants (n=98) completed the ASQ again after 3-month period. The stability coefficient of the attachment scales was calculated by using Pearson correlations (P<0.001). These coefficients were 0.625, 0.685, 0.777, and 0.605 for secure, fearful, preoccupied and dismissing respectively.

*Validity*

Convergent Validity: the Pearson correlation coefficients have been shown in table 3. Confidence factor of the Feeney’s questionnaire and secure style, are highly positively correlated. Need for approval and preoccupation factors of Feeney’s questionnaire had highly positive correlation with preoccupied style. Relationship as secondary had a positive correlation with dismissing style and a negative relationship with secure one. In addition, discomfort factor was positively correlated with fearful style. Secure styles in both questionnaires are positively correlated with each other. However, they negatively correlated with anxious-ambivalent and avoidance factors of RQ.

**Table 3:** The correlation coefficients between subscales of ASQ-24 with ASQ and RQ

**ASQ**	**ASQ-40(Feeney et al.)**					**RQ**		
	**Confidence**	**Need for approval**	**Preoccupation**	**Relationship as secondary**	**Discomfort**	**Avoidance**	**Anxious -ambivalence**	**Secure**
1- Secure	0.608^*^	0.006	-0.197^*^	-0.102^*^	-0.393^*^	-0.228^*^	-0.130^*^	0.125^*^
2- Fearful	-0.134^*^	0.235^*^	0.443^*^	0.264^*^	0.624^*^	0.362*	0.295^*^	-0.078*
3- Preoccupied	-0.110^*^	0.591^*^	0.631^*^	0.048	0.348^*^	0.047^*^	0.329^*^	-0.016
4- Dismissing	0.114^*^	-0.061	0.036	0.270^*^	0.221^*^	0.200^*^	0.036	-0.012

With the help of MANCOVA, we tried to evaluate the relationship between the different attachment styles and variables such as sex, level of education, level of parent’s education, birthplace, and marital status. We controlled the effect of age by regarding it as a covariate. The results are shown in table 4. The analysis represented significant overall effect of sex (Wilks Λ=0.981, η^2^=0.020, F (4,719)=3.57, P=0.007), univariate protected F-tests with Bonferroni corrections indicated that the mean score of fearful in women was higher than that of men (P=0.001). In subjects with academic education (exact test of Wilks Λ=0.963, η^2^=0.031, F (8, 1438)=3.41, P=0.001), secure score (P=0.015) and dismissing score (P=0.005) were higher than those having less than twelve years of education and fearful and preoccupied scores were lower. Married participants (Wilks Λ=0.987, η^2^=0.014, F (4,719)=0.244, P=0.045) had a higher secure score (P=0.045). Fathers’ education (Wilks Λ=0.987, η^2^=0.014, F (8, 1438)=1.60, P=0.071), mothers’ education (Wilks Λ=0.992, η^2^=0.009, F (8, 1438)=1.21, P=0.180) and birthplace (Wilks Λ=0.996, η^2^=0.004, F (4,719)=0.755, P=0.555) showed no significant relation with attachment styles.

**Table 4:** Comparison of Means (SD) of variables in Attachment Styles dimensions by controlling age

**Variables**		**N**	**Secure**	**Fearful**	**Preoccupied**	**Dismissing**
Sex	Male	258	4.30±0.76	3.32±0.97	3.47±0.83	3.90±0.87
	Female	476	4.37±0.74	3.52±1.08	3.61±0.98	3.89±0.92
	P. V		0.263	0.006	0.107	0.605
Education levels	<12	133	4.18±0.78	3.54±01.05	3.63±0.96	3.76±0.94
	12	352	4.37±0.76	3.54±1.03	3.65±0.91	3.88±0.90
	>12	251	4.40±0.72	3.27±1.04	3.39±0.94	3.97±0.88
	P. V		0.0190	0.025	0.004	0.041
Marital status	Single	352	4.29±0.75	3.47±1.06	3.62±0.96	3.92±0.90
	Married	384	4.39±0.75	3.42±1.02	3.52±0.91	3.87±0.91
	P. V		0.044	0.082	0.132	0.089
Birth place	Urban	659	4.36±0.75	3.43±1.05	3.57±0.94	3.88±0.90
	Rural	77	4.24±0.74	3.61±0.98	3.53±0.92	3.98± .93
			0.232	0.254	0.734	0.425
Father education	<12	426	4.33±0.73	3.58±1.05	3.63±0.95	3.92±0.91
	>12	309	4.37±0.78	3.27±1.01	3.47±0.90	3.85±0.89
	P. V		0.029	0.032	0.065	0.612
Mather education	<12	476	4.37±0.72	3.54±1.05	3.60±0.95	3.94±0.90
	>12	260	4.30± 0.79	3.27±1.01	3.50±0.90	3.79±0.90
	P. V		0.016	0.409	0.880	0.057

**Figure 1:**
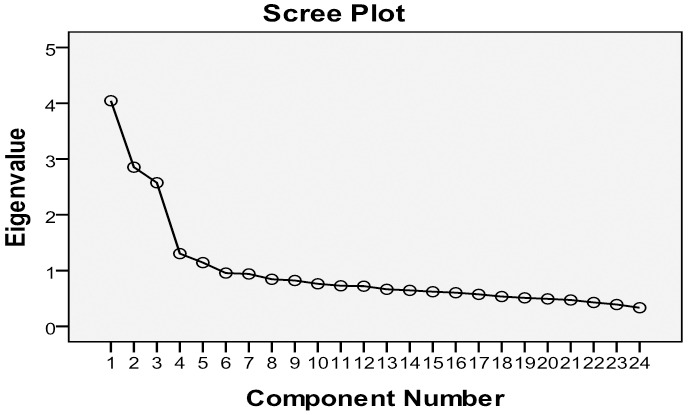
The Scree plot of principle component

Construct validity: Bartlett’s test of sphericity demonstrates the appropriateness of applying factor analysis of our data set (KMO=0.817, P<0.0001). Explanatory factor analysis (rotation method: oblimin with Kaiser Normalization, extraction method: principal components analysis) showed four extracted factors with eigenvalues greater than 1.2, as 4.05, 2.86, 2.58 and 1.31 respectively. Similarity, the scree plot (figure 1) also recommended four component principles, which have eigenvalues greater than 1.2. The first four factors explained 44.94% of total variance. Table 5 shows that all factors have reasonably high factor loadings. The first column of this table contains ASQ’s items.

**Table 5:** The components loading, eigenvalues, percent of variance and communality of 4-factor in ASQ-24

**Items**	**Factor 1**	**Factor 2**	**Factor 3**	**Factor 4**	**Communality**
Q4-I’m afraid that my hopes will be deceived when I get too closely related to others	0.732				0.557
Q2-I would like to be open to others, but I feel I cannot trust other people.	0.726				0.526
Q21-I feel uncomfortable when relationships with other people become close.	0.649				0.434
Q18-I am wary to get engaged in close relationships because I’m afraid to get hurt.	0.647				0.482
Q8-I have the impression that usually I like others better than they like me.	0.507	0.209			0.322
Q3-I would like to have close relationships with other people, but I find it difficult to fully trust them.	0.378	0.199			0.413
Q7-I avoid close ties.	0.358	0.236	0.289	0.275	0.373
Q13-I feel at ease in intimate relationships.		0.701			0.500
Q12-I find it easy to get engaged in close relationships with other people.	0.168	0.626	0.165		0.434
Q9-I trust other people and I like it when other people can rely on me.		0.586		0.251	0.376
Q14-I like to be self-sufficient.	0.398	0.562			0.499
Q16-I think it is important that people can rely on each other.	0.255	0.561	0.269		0.447
Q11-It is important to me to be independent.	0.312	0.502	0.222		0.406
Q1-I feel at ease in emotional relationships		0.475		0.258	0.319
Q20-I trust that others will be there for me when I need them.	0.312	0.347			0.212
Q17-I don’t worry about being alone: I don’t need other people that strongly			0.770		0.586
Q24-I feel comfortable without having close relationships with other people		0.245	0.568		0.400
Q15-I don’t worry whether people like me or not			0.539	0.237	0.387
Q5-I prefer that others are independent of me, and that I am independent of others	0.381		0.430		0.375
Q10-I am often afraid that other people don’t like me				0.748	0.639
Q6-I often wonder whether people like me				0.707	0.617
Q19-I usually find other people more interesting than myself				0.704	0.486
Q23-I fear to be left alone				0.476	0.491
Q22-I find it important to know whether other people like me				0.439	0.507
Eigenvalue	4.05	2.86	2.58	1.31	
% of variance	16.86	11.9	10.74	5.44	

The first factor in the Persian-adapted ASQ (P.A.ASQ) included five items of the original fearful factor, one item of preoccupied style: “*I have the impression that usually I like others better than they like me*” and, one item of secure style: “*I avoid close ties*”. The item “*I have the impression that usually I like others better than they like me*” was not able to specify a style in our sample and thus was removed. This item originally belonged to the preoccupied factor in the ASQ; but in the P.A.ASQ it was loaded in the first factor (fearful) and with a smaller factor load in the second factor (secure). Therefore, it seems that this item fails to identify the preoccupied style in the Iranian culture.

The item *“I avoid close ties*” was originally with a negative coefficient belonged to the secure factor. However, in our model, this item with loading of 0.36 was loaded in the first factor (fearful) and it was located in the second factor (secure) with a smaller negative loading factor. Therefore, regarding its origin, we put it in the secure factor.

Therefore, the appropriate title for this factor is fearful. The second factor was named secure because it contained six items of the original secure factor and two items of dismissing: “*It is important to me to be independent*” and “*I like to be self-sufficient*”. Loading of these two items in this factor is probably due to misperception and cultural differentiation. Accordingly, we decided to locate them in the original place after changing the concept of these items.

Regarding the item, “*I like to be self-sufficient*”; it seems that “*self-sufficient*” have a different resonance in the mind of the Iranian people. In this culture, it means self-reliant and independent rather than dismissing. Instead, we used the term “stubborn”, “bullheaded”. About the item “*It is important to me to be independent*”, we changed it to the sentence “*the need and dependence to others bothers me*” and they were both placed in their original factor, dismissing. Following these modifications, the Cronbach’s alpha for the dismissing factor increased from 0.588 to 0.688.

The third factor named “dismissing”, includes three items of its original subscale and one item “*I don’t worry whether people like me or not*” of preoccupied. It had a negative coefficient in original subscale, but it seems that its consideration with a positive coefficient loading in this factor is reasonable for our sample. The last factor contains all original items of preoccupied.

By factor analysis, we reached the four factors similar to the study by Van Oudenhoven.^21^ These factors are in accordance with the four factors discussed by Bartholomew and Horowitz. Furthermore, we used forced-explanatory factor analysis (quartimax rotation principal components analysis by two factors).The first factor embraced items 4, 18, 2, 10, 6, 21, 3, 23, 8, 7, 19, 5 and 22. Indeed, this component (anxiety) includes two factors: fearful and preoccupied and the second factor includes items 13, 14, 12, 11, 16, 1, 9, 24, 20, 15 and 17. Dismissing and secure styles were loaded in the second component (avoidance).

## Discussion

The present research was conducted to determine the reliability and validity of the Persian (Farsi) version of adult attachment styles. The reliability of the secure, fearful, and preoccupied scales ranges from reasonable to good. However, the internal consistency of the dismissing scale is moderate (Cronbach’s α=0.588) which might be due to the confusion of respondents by the concepts of the questions in this category. This may be due to the ambiguity of the concept of “dismissing” and its different meaning in different cultures. Individuality and independence are positive characteristics in western culture. However, these attributes have a negative connotation in some eastern cultures. A person is dependent to his/her family both financially and emotionally. Contrary to the individualistic worldview of the people in western cultures, dependence is not equal to separation from family and living alone in eastern cultures. It seems that the terms “self-sufficient” and “independent” are confusing to the Iranian people. The low internal consistency of this item may be due to this difference. Van Oudenhoven et al. pointed out the low reliability of this item and mentioned that it can explain the weak relations between dismissing attachment and other variables.^24^ Montoliva et al. found a difference between men and women in dismissing attachment regarding their attitude in a romantic relationship. Perhaps men and women can relate to a different interpretation of this item.^28^ By changing the two items “*It is important to me to be independent*” and “*I like to be self-sufficient*” to “*the need and dependence to others bothers me”* and “*I like to be stubborn; bullheaded*” respectively, the Cronbach’s alpha coefficient was increased to 0.688. It supports the assumption that the participants were not able to grasp the meaning of these items. The cumulative variance of the four factors was 0.45 (table 5) which can be attributed to the different meaning of attachment concepts in various cultures. Considering the period between the two measurements (about three months), the stability coefficients are reasonably high. Shaver and Hazan state that “more than one longitudinal study has approximately found 80% stability over several years in economically stable samples”.^29^ The stability was the highest for the preoccupied attachment style. Brennan, Clark, and Shaver,^30^^,^^31^ suggested that adult attachment had better be assessed by measuring two underlying factors namely; anxiety and avoidance. Most self-report instruments identified only anxious and avoidant attachment dimensions.^30^ These two items differentiate individuals based on the need for company and dependence. The attachment scales of the ASQ correlated highest with the corresponding vignette of the RQ and the Feeney questionnaire.^22^ The correlations were moderate and the differences between correlations were not impressive between ASQ and RQ.

Generally, women and men differ on relational variables. Therefore, it is not surprising that we found gender differences with respect to the attachment styles. This study showed higher scores on fearful attachment style in women, indicating women are more concerned about being admired by other people. In traditional societies such as Iran, women are encouraged not to display emotions and they have more social inhibitions than men do. Previous studies showed that the dismissing attachment is more prevalent in men than women.^30^^,^^32^ As noted by Cross and Madson,^33^ this may indicate that women are more socially oriented than men. However, our study showed no difference between them in terms of dismissing style. Social prohibitions may play a role, which thwarts the women to play an active role in social interactions. Further and more extensive studies are required to address this issue.

Schmitt reported that in East Asian collective societies, which individuals strive for approval by others, preoccupied style is more frequent.^34^ Also, Van Oudenhoven pointed out that women are more preoccupied.^24^ The fact that they are fearful in the Iranian society might be rooted in the social and religious variables, which create a different, and a discriminating environment for girls. As could have been predicted, more educated and married individuals have higher scores in secure style. As noted by Van Oudenhoven, further studies have to address the issue of low consistency of dismissing scale and the ambiguity of some items related to fearful style. In addition, more research is required to evaluate the relations among the four-attachment styles.^24^

## Conclusion

In this study, a relatively valid questionnaire to measure attachment style aiming the Iranian population was developed. Factor analysis showed the need to revise a few items to fit the Iranian population. Generally, the Persian version of the ASQ showed a reasonable reliability and validity. Following certain modifications, this questionnaire can be deployed in the Iranian society. This study paves the way for a wider study in different cultural groups in our society. This would challenge the findings of this study leading to a higher level of validity and reliability of the questionnaire 
